# Developmental Proteomics Reveals the Dynamic Expression Profile of Global Proteins of *Haemaphysalis longicornis* (Parthenogenesis)

**DOI:** 10.3390/life15010059

**Published:** 2025-01-06

**Authors:** Min-Xuan Liu, Xiao-Pei Xu, Fan-Ming Meng, Bing Zhang, Wei-Gang Li, Yuan-Yuan Zhang, Qiao-Ying Zen, Wen-Ge Liu

**Affiliations:** 1 College of Veterinary Medicine, Gansu Agricultural University, Lanzhou 730070, China; mxliu1991@163.com; 2 State Key Laboratory Animal Disease Control and Prevention, Key Laboratory of Veterinary Parasitology of Gansu Province, Lanzhou Veterinary Research Institute, Chinese Academy of Agricultural Science, Lanzhou 730046, China; 3 Key Laboratory of Forensic Medicine, Institute of Medical Sciences, School of Basic Medical Sciences, Xinjiang Medical University, Urumqi 830011, China; 19990224556@163.com (X.-P.X.); mengfanming1984@163.com (F.-M.M.); ppag98761695535e@sohu.com (B.Z.); 4 Animal Husbandry and Veterinary Station of Zhenyuan County, Zhenyuan 744500, China; 18719718116@163.com (W.-G.L.); 18294092571@163.com (Y.-Y.Z.)

**Keywords:** *Haemaphysalis longicornis*, proteomic, development, iTRAQ, differential protein

## Abstract

*H. longicornis* is used as an experimental animal model for the study of three-host ticks due to its special life cycle and easy maintenance in the laboratory and in its reproduction. The life cycle of *H. longicornis* goes through a tightly regulated life cycle to adapt to the changing host and environment, and these stages of transition are also accompanied by proteome changes in the body. Here, we used the isobaric tags for a relative and absolute quantification (iTRAQ) technique to systematically describe and analyze the dynamic expression of the protein and the molecular basis of the proteome of *H. longicornis* in seven differential developmental stages (eggs, unfed larvae, engorged larvae, unfed nymphs, engorged nymphs unfed adults, and engorged adults). Gene Ontology (GO) and Kyoto Encyclopedia of Genes and Genomes (KEGG) analysis of the differentially expressed proteins (DEPs) were used. In our study, A total of 2044 proteins were identified, and their expression profiles were classified at different developmental stages. In addition, it was found that tissue and organ development-related proteins and metabolism-related proteins were involved in different physiological processes throughout the life cycle through the GO and KEGG analysis of DEPs. More importantly, we found that the up-regulated proteins of engorged adult ticks were mainly related to yolk absorption, degradation, and ovarian development-related proteins. The abundance of the cuticle proteins in the unfed stages was significantly higher compared with those of the engorged ticks in the previous stages. We believe that our study has made a significant contribution to the research on *H. longicornis*, which is an important vector of SFTSV. In this study, we identified changes in the proteome throughout the *H. longicornis* development, and functional analysis highlighted the important roles of many key proteins in developmental events (ovarian development, the molting process, the development of midgut, the development and degeneration of salivary glands, etc.). The revelation of this data will provide a reference proteome for future research on tick functional proteins and candidate targets for elucidating *H. longicornis* development and developing new tick control strategies.

## 1. Introduction

Ticks are globally distributed, blood-sucking ectoparasites that are related to tick-borne diseases, such as protozoan parasites (*Babesia*), viruses (Tick-borne Encephalitis Virus), Lyme disease (*Borrelia*), and mycoplasmas (*Chlamydia psittaci*). The impact of tick-borne diseases is staggering, especially in developing countries, where tick-borne diseases affect about 80% of the world’s cattle, with economic losses estimated to be between USD 13.9 billion and USD 18.7 billion [1,2].

*H. longicornis* is an important species of ticks, with a four-stage life cycle: egg, larva, nymph, and adult [3]. In particular, larvae, nymphs, and adults need to be parasitic on the host, sucking blood to obtain nutrition and energy reserves, and then undergo molting as they proceed to the next stage or lay eggs. In the life cycle of *H. longicornis*, which spans for about four months, the body shape of *H. longicornis* will change greatly as well as many organs and tissues, which are all accompanied by the rise and fall of gene expression in *H. longicornis* [4]. The differential expression of these proteins in specific periods provides the molecular basis and guarantee condition for the maintenance and body changes of *H. longicornis* in a specific period.

In previous studies, two-dimensional electrophoresis (2-DE) construction, time-of-flight mass spectrometry (MALDI-TOF-MS), and two-dimensional differential gel electrophoresis (2D-DIGE) combined with MALDI-TOF-MS have been used in tick studies. In addition, a lot of proteins in the tick midgut, salivary gland, and ovary and a variety of tick-related proteins have been successfully identified [5,6,7].

The isobaric tags for relative and absolute quantification (iTRAQ) technology is an advanced and accurate protein sequencing technology, which can determine the protein components in a sample more accurately and comprehensively as compared to traditional methods. A precise mass spectrometry detection method is an important prerequisite for comparative proteomics analysis, and as a quantitative proteomics method, iTRAQ can generate information about the abundance of hundreds of proteins at a time. In addition, it allows repeated multiplexing of parallel biology or techniques (eight iTRAQ markers) in liquid chromatography with tandem mass spectrometry (LC-MS)/MS experiment, thus overcoming the difference between measurements in a single MS-based shot bullet spectrum analysis [8,9,10].

At present, iTRAQ has been successfully applied to the study of differentially expressed proteins (DEPs) in the salivary glands of *H. longicornis*. Researchers found that the expression of some proteins related to energy production was continuously down-regulated during salivary gland degeneration, while some proteins related to DNA or protein degradation were up-regulated through this method. In addition, the expression of some proteins related to apoptosis or autophagy also changed [11,12].

However, there is still no study that confirms and analyzes the overall protein expression pattern of *H. longicornis* at the whole developmental stages. Therefore, the purpose of this study was to use eight-fold iTRAQ markers and LC-MS/MS to further understand the functional differences between different stages of the life cycle of *H. longicornis*, more importantly, to identify and quantify the protein content in these stages, and to find out the key proteins in the development of *H. longicornis*. This study not only provides a comprehensive and reliable data platform for tick prevention and control but also provides more potential targets for the development of tick vaccines.

## 2. Materials and Methods

### 2.1. Rabbits and Parasites

The clean New Zealand white rabbit was obtained from the Experimental Animal Center of the Lanzhou Veterinary Research Institute. The *H. longicornis* isolate used was the monoclonal strain of clean *H. longicornis*, which was cultivated and preserved by the Lanzhou Veterinary Research Institute [13].

### 2.2. The Collection of H. longicornis at Different Developmental Stages

Referring to the method in a previously described study [13], ticks in different unfed stages (unfed larvae, unfed nymphs, and unfed adults) were placed on clean New Zealand white rabbits until they were engorged. Afterward, the ticks were collected into breathable glass tubes and placed in an incubator with a humidity of 80% and a temperature of 27 °C, waiting for them to enter the next life stage, i.e., molting or spawning.

In short, seven different developmental stages of *H. longicornis* (parthenogenesis) were obtained: 500 eggs within 24 h after spawning, 500 unfed larvae within 24 h after hatching, 300 engorged larvae that fell off within 24 h after being blood fed, 300 unfed nymphs within 24 h after molting, 30 engorged nymphs that fell off within 24 h after being blood fed, 30 unfed adults within 24 h after molting, and 3 engorged adults that fell off within 24 h after being blood fed. All samples were washed in bromogeramine, alcohol, and deionized water successively. After that, the samples were placed in an Eppendorf tube in triplicate and preserved at –80 °C.

A single sample from each development stage was divided into three LC-MS/MS runs (technical replicates), with no biological replicates due to each sample set containing mixed samples.

### 2.3. Protein Extraction

Protein extraction was performed according to Wang’s research with some adjustments [11]. About 2 g from each sample was put into the 1.5 mL centrifuge tube, and a 5 mm magnetic bead and an appropriate amount of Lysis Buffer 3 were added, respectively. Then, ethylenediaminetetraacetic acid (EDTA) was added, the sample with a final concentration of 1 mM was vortexed, and the tube was allowed to rest for 5 min. Afterward, dithiothreitol (DTT) was added, for a final concentration of 10 mM, and was shaken with a tissue grinder for 2 min (power, 50 Hz; time, 120 s). The supernatant was centrifuged at 25,000× *g* at 4 °C for 20 min, and the final concentration was 10 mM. The tube was then placed in a 56 °C water bath for 1 h. Then, iodoacetamide (IAM) was added to a final concentration of 55 mM at room temperature (at about 22–25 °C) and was allowed to stand for 45 min. Acetone, at a volume four times that of the IAM, was added, and the solution was allowed to stand at −20 °C for 2 h. The steps were repeated three times until the supernatant was colorless. Then, the solution was centrifuged at 25,000× *g* and 4 °C for 20 min, and the supernatant was discarded. Afterward, a 5 mm magnetic bead was added as well as a proper amount of Lysis Buffer 3 to allow the solution to precipitate. Then, the solution was shaken for 2 min with a tissue grinder (power, 50 Hz; time, 120 s) and centrifuged at 25,000× *g* 4 °C for 20 min, and the supernatant was collected for quantitative analysis.

### 2.4. Proteolysis and Peptide Labeling

Protein solution (100 μg) was taken from each of the seven samples; then, 2.5 μg of the trypsin enzyme was added at a protein-to-enzyme ratio of 40:1, and the solution was hydrolyzed at 37 °C for 4 h. Then, trypsin was added according to the above ratio, continuing the enzymatic hydrolysis at 37 °C for 8 h, and then we desalinated the hydrolyzed peptides using a Strata X column (Phenomenex, Torrance, CA, USA), and they were vacuum dried. Peptide labeling was performed by iTRAQ Reagent 8-plex Kit (AB Sciex, Framingham, MA, USA) according to the manufacturer’s protocol. After the reagent was restored to room temperature, 50 μL of isopropanol was added to each tube. After vortex shock, the peptide sample was dissolved with 0.5 M tetraethylammonium bromide (TEAB) and added to the corresponding iTRAQ labeling reagent. Different sample peptides were labeled with different iTRAQ tags, and the solution was allowed to stand at room temperature for 2 h.

### 2.5. LC-MS/MS Analysis

The sample was separated by a Shimadzu LC-20AB liquid phase system (Shimadzu, Kyoto, Japan) with a 5 µm, 250 × 4.6 mm Gemini C18 column (Phenomenex, Torrance, CA, USA). The dried peptide sample was redissolved and injected with 2 mL of mobile phase A (5% ACN pH 9.8) and eluted at a flow rate gradient of 1 mL/min: 5% mobile phase B (95% acetonitrile [can], pH 9.8) for 10 min, 5% to 35% mobile phase B for 40 min, 35% to 95% mobile phase B for 1 min, mobile phase B for 3 min, and 5% mobile phase B for 10 min. The elution peak was monitored at a 214 nm wavelength, and one component was collected every minute. Combined with the chromatographic elution peak map, 20 components were obtained, and then they were freeze dried.

The dried peptide sample was re-dissolved with mobile phase A (2% ACN, 0.1% FA) and centrifuged at 20,000× *g* for 10 min, and the supernatant was injected. The separation was carried out by the nanoliter liquid chromatograph of Shimadzu Company LC-20AD (Shimadzu, Kyoto, Japan). The sample was first enriched and desalted in a trap column and then connected in series with a self-assembled C18 column (75 μm inner diameter, 3.6 μm column size, 15 cm column length). The separation was carried out at a flow rate of 300 nL/min through the following effective gradient: 0–8 min, 5% mobile phase B (98% ACN, 0.1% formic acid [FA]); 8–43 min, mobile phase B increased linearly from 8% to 35%; and 43–48 min, mobile phase B increased from 35% to 60%. In 48–50 min, the mobile phase B rose from 60% to 80%; in 50–55 min, the mobile phase B rose to 80%, the mobile phase B, 55–65 min, and 5%, the mobile phase B. The end of the nanoliter liquid phase separation was directly connected to the mass spectrometer.

The peptides separated by liquid phase were ionized by a nanoESI source (Agilent Technologies, Santa Clara, CA, USA), and then they were placed into a tandem mass spectrometer Q-Exactive HF (Thermo Fisher Scientific, San Jose, CA, USA) for data-dependent acquisition (DDA) mode detection. The main parameters were as follows: the ion source voltage was set to 1.6 kV; the scanning range of the first-stage mass spectrometry was 350~1600 *m*/*z*; the resolution was set to 60,000 m; the initial m-stroke z of the second-stage mass spectrometry was fixed to 100; and the resolution was 15,000. The screening conditions for the parent ions of the secondary fragmentation were as follows: the charge was 2+ to 6+, and the parent ions with a peak intensity of more than 10,000 were in the top 30. The ion fragmentation mode was higher-energy C-trap dissociation (HCD), and fragment ions were detected in Orbitrap. The dynamic exclusion time was set to 30 s, and the automatic gain control (AGC) was set to first-level 3E6 and second-level 1E5.

### 2.6. Database Search and Bioinformatics Analysis

The original MS data were converted into a general format (.mgf) file with Proteome Discoverer 1.4, then the data file was used to query the tick database in the NCBI “https://www.ncbi.nlm.nih.gov/protein/?term=tick (accessed on 15 April 2020)”and the 813103 amino acid sequences of annotated tick proteins were used to compare the original sequences. ProteinPilot protein software 4.5 (AB SCIEX) was used for further identification and quantification of proteins. In order to filter the results, we used an error detection rate of less than 0.01 for the identification, and for the quantification, a confidence level of 95% or an unused confidence score greater than 1.3a was used. For the DEPs, values | log^2^-fold change | > 1 were regarded as up-regulated or down-regulated proteins, respectively. Finally, the MS data have been deposited in iProX (Integrated Proteome Resources, “http://www.iprox.org/ (accessed on 19 April 2020)”.

Functional classification of the DEPs was carried out according to GO http://www.geneontology.org (accessed on 20 April 2020) annotation and analysis. The DEPs were divided into three categories, namely, molecular function, biological processes, and cellular components. The KEGG http://www.kegg.jp/kegg/ (accessed on 20 April 2020) was used to predict the molecular functions, biological processes, and important DEP pathways.

### 2.7. Quantitative Reverse Transcription Polymerase Chain Reaction (RT-qPCR) Analysis

Six proteins (CRK, flotillin, Mo-25, dystrophin, septin-1, and septin-2) were randomly selected from the protein library built by iTRAQ, and these genes were compared at the transcriptional level by RT-qPCR. The first cDNA strand was synthesized using the total RNA from the samples from seven stages (egg, unfed larva, engorged larva, unfed nymph, engorged nymph, unfed adult, and engorged adult) using the PrimeScript Reverse Transcriptase kit (TaKaRa, Dalian, China), according to the manufacturer’s instructions. The primers of the six genes used in the experiment were all designed by our laboratory, and the amplification efficiency of the primers was evaluated to be more than 90%. The RT-qPCR was carried out in accordance with the methods described earlier, and relative transcript levels were calculated using the 2^−ΔΔCt^ method [13]. The information on the primers of the selected gene and the reference gene beta-actin is shown in Table 1.

### 2.8. Statistical Analysis

All statistical data were processed with GraphPad Prism software version 7 (GraphPad, San Diego, CA, USA), and the data were analyzed by Student’s *t*-test. *p* < 0.05 was considered statistically significant.

## 3. Results and Discussion

The development of *H. longicornis* is accompanied by many changes in the host, such as sucking blood, molting, and spawning. In the whole process, the occurrence, development, and degeneration of tissues and organs are also accompanied by changes in the protein profile. The lack of a detailed and comprehensive protein dynamic spectrum during the development of *H. longicornis* limits the research on the development of *H. longicornis*, the screening of drug resistance genes, and the development of anti-tick drugs and other related studies. The iTRAQ quantitative proteomics is a powerful and novel tool for characterizing protein changes among different samples, which has been well verified in a variety of organisms and helps people to analyze the protein profiles of many organisms under different conditions and different developmental stages [14,15,16]. Therefore, in this experiment, in order to clearly understand the differences in the physiological functions of *H. longicornis* at different developmental stages and, especially, in order to identify, quantify, and compare the protein expression profiles of *H. longicornis* at different developmental stages, we used iTRAQ quantitative proteomics technology to analyze the related functions of *H. longicornis* proteins at different developmental stages.

### 3.1. Overview of Main Data and Protein Identification

**Figure 1:**
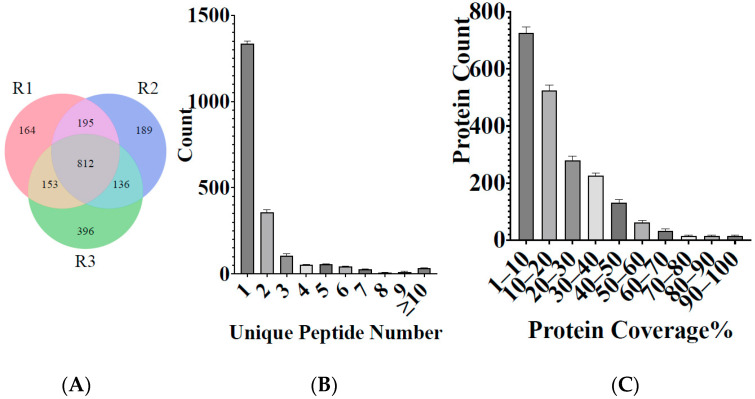
(**A**) Wayne diagram of the total proteins identified by three repeated experiments on *H. longicornis*. R1, repeat 1; R2, repeat 2; and R3, repeat 3. (**B**) Distribution of the specific peptides and (**C**) protein coverage distribution.

In this study, iTRAQ was used to identify the proteomic at different stages of the life cycle of *H. longicornis*, that is, the egg, unfed larva, engorged larva, unfed nymph, engorged nymph, unfed adult, and engorged adult. In the three repeated experiments, a total of 2044 proteins were identified after eliminating several potentially contaminating proteins, from 4405 peptides, which were matched with 2,608,862 spectra at a false discovery rate of 1% (Table 2, Supplementary Table S1). As shown in Figure 1A, the number of proteins identified in the three repeated experiments was 1353, 1323, and 1437, respectively, while 812 proteins were identified to be shared in the three repeats. Most of the proteins were identified by one peptide; specifically, 1336.3 ± 51.47 (65.22%) proteins were identified based on one peptide. More than 357.7 ± 11.43 (17.50%) proteins were identified based on two peptides, and 103 ± 5.41 (5.02%) proteins were identified based on three peptides (Figure 1B).

The sequence coverage of a specifically identified protein is estimated as the percentage of matching amino acids between the identified peptides with more than 95% confidence divided by the total number of amino acids in the protein sequence. The sequence coverage of 725.43 ± 52.69 (35.49%) proteins was less than 10%, while the sequence coverage of 527 ± 29.64 (25.72%) proteins was 10–20%. The MS data have been deposited in China National Center for Bioinformation “https://ngdc.cncb.ac.cn/omix/release/OMIX707 (accessed on 25 October 2021)” (Figure 1C).

### 3.2. Protein Quantification

**Figure 2:**
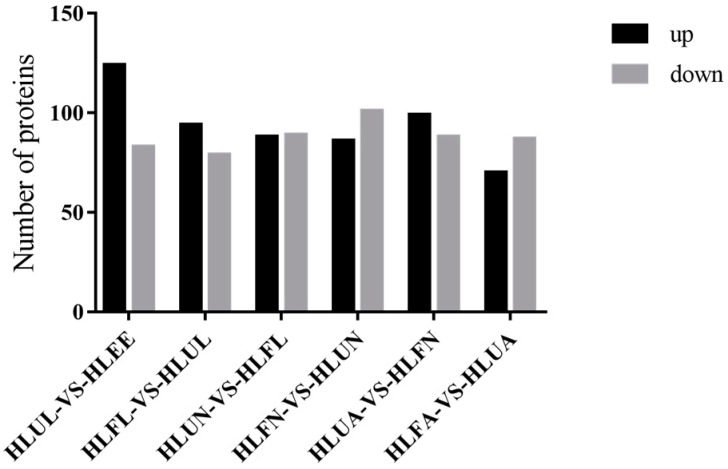
Comparative analysis of the differentially expressed proteins (DEPs) in the different developmental stages of *H. longicornis*. EE, egg; UL, unfed larva; FL, engorged larva; UN, unfed nymph.

The *H. longicornis* tick has a complicated life cycle, and the molecular basis of its growth and development is still poorly understood. In order to study the protein profile during its growth process, we have obtained samples from seven differential development stages (egg, unfed larva, engorged larva, unfed nymph, engorged nymph, unfed adult, and engorged adult). A proteomic analysis was performed, and the proteins with |log^2^-fold change|> 1 and *p* < 0.05 were considered DEPs. A pairwise comparison of the proteins of each successive stage was conducted, and it was found that, compared with those of eggs, the unfed larvae had 124 up-regulated proteins and 83 down-regulated proteins; compared with those of hungry young ticks, there were 94 up-regulated proteins and 79 down-regulated proteins in the engorged larvae; compared with the engorged larvae, the unfed nymphs had 88 up-regulated proteins and 89 down-regulated proteins; compared with unfed nymphs, there were 86 up-regulated proteins in the ticks and 101 down-regulated proteins in the engorged nymphs; compared with engorged nymphs, there were 99 up-regulated proteins and 88 down-regulated proteins in the unfed adults; and compared with unfed adults, there were 70 up-regulated proteins and 87 down-regulated proteins in the engorged adults. Figure 2 shows the number of DEPs at different developmental stages.

### 3.3. RT-qPCR Analysis

**Figure 3:**
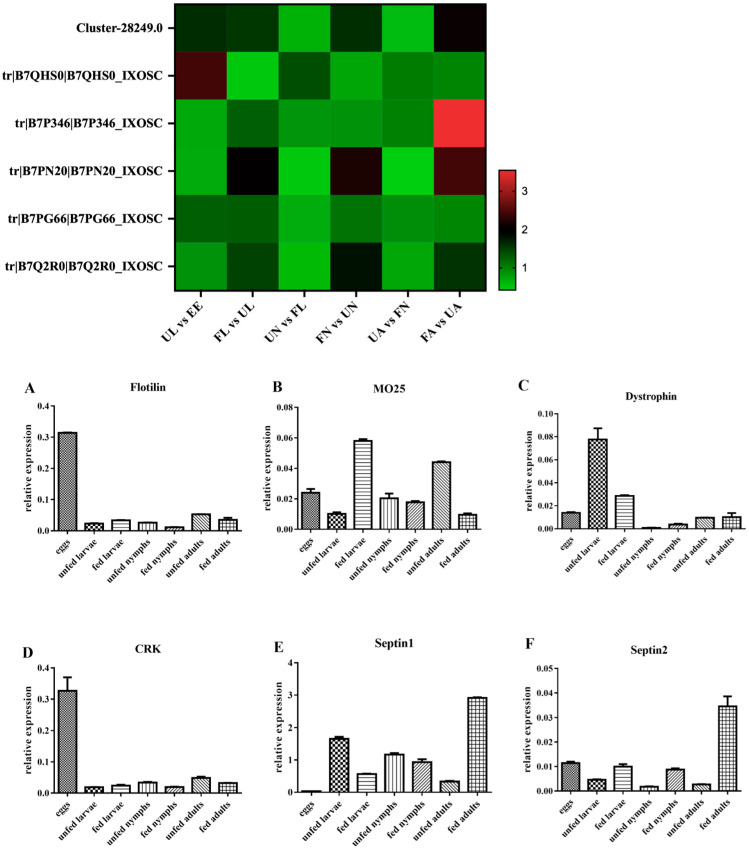
The heat map represents the proteome analysis results of six genes compared across different developmental stages, while the bar graph of (**A**–**F**) displays the RT-qPCR analysis results for CRK, flotillin, Mo-25, dystrophin, septin-1, and septin-2, respectively). EE, egg; UL, unfed larva; FL, engorged larva; UN, unfed nymph; FN, engorged nymph; UA, unfed adult; FA, engorged adult.

The transcriptional levels of six genes (CRK, flotillin, Mo-25, dystrophin, septin-1, and septin-2) were examined at seven stages. Through the analysis of the results, we found that the change in their transcriptional levels was not consistent with the trend of protein levels in iTRAQ at different developmental stages (Figure 3).

The reason for this result is mainly attributed to the fact that the RNA level is only a moderate proxy for protein abundance and does not fully represent the protein expression abundance. These results highlight that it is necessary to analyze the differentiation mechanism components of *H. longicornis* at the protein level, which are involved in basic biological processes such as signal transduction, substance transport, catalytic activity, metabolism, and so on.

### 3.4. Expression Profile of the Identified Proteins

#### 3.4.1. Chitin-Binding Proteins

**Figure 4:**
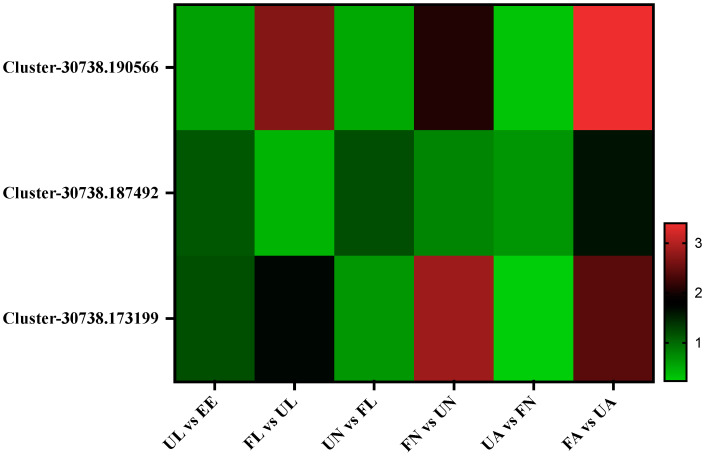
Chitin-binding proteins. EE, egg; UL, unfed larva; FL, engorged larva; UN, unfed nymph; FN, engorged nymph; UA, unfed adult; FA, engorged adult.

In this experiment, three chitin-binding proteins (Cluster-30738.173199, Cluster-30738.190566, and Cluster-30738.187492) were identified. Among them, the two peritrophic membrane chitin-binding proteins shared the same expression trend; that is, they were decreased in the process of an egg hatching into an unfed larva and then increased significantly with blood sucking. On the other hand, in the process of entering the next stage after blood sucking, it showed a significant downward trend and then reached a peak in the engorged adult (Figure 4).

The peritrophic membrane (PM) is an important organ of blood-sucking arthropods, which provides protection for the microvilli of digestive tract epithelial cells and is a sturdy barrier to protect the intestinal tract from physical damage caused by the structure of food intake and the invasion of parasites and other pathogens [17]. Moreover, many studies have used histochemical and biochemical techniques to show the presence of chitin on the perineal membrane [18,19]. A previous study found that the PM of *H. longicornis* was significantly different between the larvae and the adult stages, and the presence of PM chitin-binding proteins was observed [20]. Similarly, in this study, we identified two kinds of PM chitin-binding proteins, both of which showed the same upward trend in the process of blood intake, which is consistent with previous studies, and can also explain their protective role in the process of blood uptake.

#### 3.4.2. Digestion-Related Proteins

The digestion of blood provides energy and nutrients for maintaining the growth and metabolism of ticks, which is a complex process, requiring the cooperation of a variety of proteins to process and deal with the hemoglobin ingested and then convert it into the tick’s own nutrients [21]. In this study, a variety of proteins related to digestion were found, which would help to use dynamic strategies to explain and clarify the blood digestion process during the development of *H. longicornis.*

**Figure 5:**
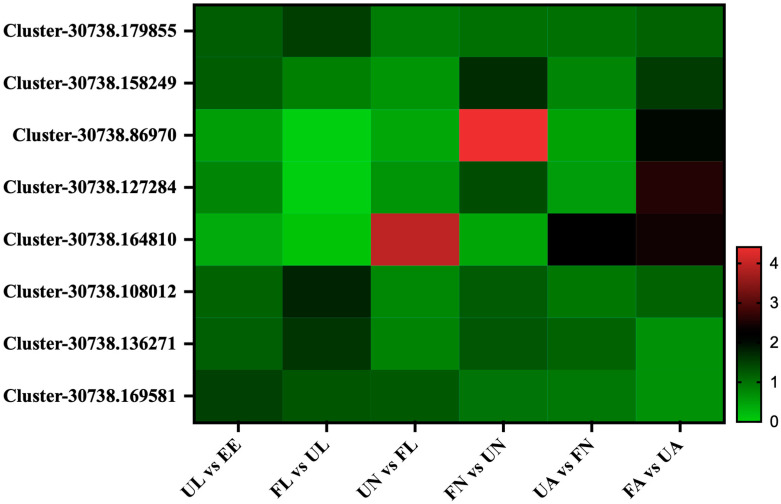
Digestion-related proteins. EE, egg; UL, unfed larva; FL, engorged larva; UN, unfed nymph; FN, engorged nymph; UA, unfed adult; FA, engorged adult.

The differences in the expression of proteins related to digestion were analyzed in the different stages of the experiment. Interestingly, we found four trypsin proteins (Cluster-30738.179855, Cluster-30738.158249, Cluster-30738.86970, and Cluster-30738.127284). They increased significantly from the unfed nymph stage to the engorged nymph stage as well as from the unfed adult stage to the engorged adult stage. In addition, we also found three carboxypeptidase proteins (Cluster-30738.164810, Cluster-30738.108012, and Cluster-30738.136271). The expression of Cluster-30738.164810 showed low abundance in both the egg and larva stages and then increased rapidly from the unfed nymph stage and lasted until the engorged adult stage. In addition, the expression abundance of two proteins (Cluster-30738.108012 and Cluster-30738.136271) increased from the egg to the larva stage. After that, they showed upward trends from unfed stages to corresponding engorged stages. Leucine aminopeptidase (Cluster-30738.169581) increased from the egg to the unfed larva stage and then began to decrease gradually and decreased to the lowest level in the unfed adult stage (Figure 5).

#### 3.4.3. Vitellogenin Proteins

**Figure 6:**
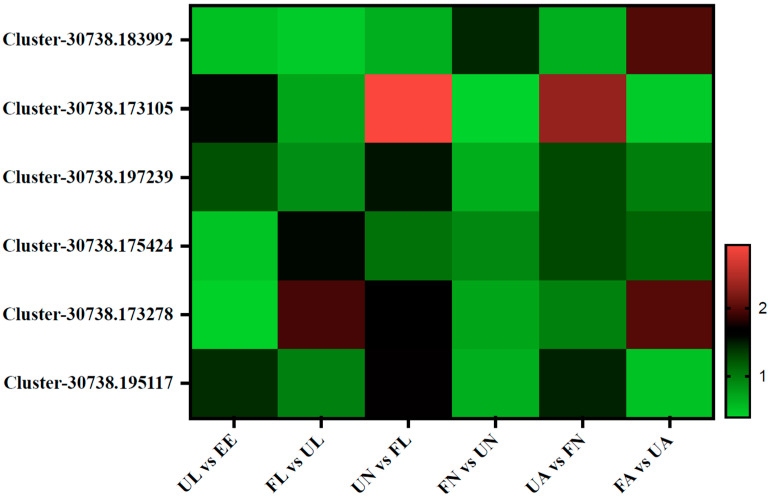
Vitellogenin (Vg)-related proteins. EE, egg; UL, unfed larva; FL, engorged larva; UN, unfed nymph; FN, engorged nymph; UA, unfed adult; FA, engorged adult.

During the development of ticks, vitellogenin (Vg) is synthesized as a high-molecular-weight precursor in body fat, the gut, and the ovary. After that, the Vg is released into the hemolymph and absorbed and accumulated by oocytes through receptor-mediated endocytosis. At this time, it is named Vt, which is an important source of nutrients for embryonic development [22,23]. In this study, six vitellogenin proteins were identified: Vg1, Vg2, Vg3, Vg4, Vg5, and Vg6 (Cluster-30738.183992, Cluster-30738.173105, Cluster-30738.197239, Cluster-30738.175424, Cluster-30738.173278, and Cluster-30738.195117). Among them, Vg2, Vg3, and Vg6 showed the same expression pattern. The expression abundance of these Vg proteins increased significantly from an egg hatching into an unfed larva but began to decrease during the development of an unfed larva to an engorged larva and increased again during molting into an unfed nymph. Then, after sucking blood in the engorged nymph stage, the content decreased again. While molting into the unfed adult stage, their content increased again, and then it declined again after the last bloodsucking in the engorged adult stage. However, the expression abundance of Vg1 increased significantly from the unfed nymph to the engorged nymph stage and from the unfed adult to the engorged adult stage. The results of the Vg4 and Vg5 showed that the expression abundance increased significantly from the unfed larva to the engorged larva stage and from the unfed adult to the engorged adult stage (Figure 6).

As early as 2010, scientists successfully annotated Vg1, Vg2, and Vg3 in *H. longicornis* and identified the protein size of these three Vgs. Also, they observed a rapid increase in Vg2 and Vg3 transcription levels in the body fat on the second day of feeding, a significant increase in Vg1 transcription in the midgut on the fourth day, and an increase in the mRNA expression of Vg2 in the ovary from the fourth day in feeding. To explore their role in the development of *H. longicornis*, through RNAi technology, it was found that the knockdown of Vgs could significantly affect the full blood weight of ticks in field teaching, and Vgs are necessary for egg weight and oviposition [23].

In 2018, scientists explored the ovariogenesis of *Boophilus microplus* and identified seven Vt peptides, which are the corresponding products of five different Vgs (Vg1, Vg2, Vg3, Vg4, and Vg5). They were observed to increase during the feeding phase, most of which increased rapidly at the end of blood feeding [24].

In this study, it was found that the six Vgs showed different expression patterns in the different developmental stages of *H. longicornis*, suggesting that they may play different roles in different tissues and physiological processes, which needs to be further explored in the future.

#### 3.4.4. Cuticle Proteins

The cuticle of ticks is an important defense tissue, which can resist bad weather and other physical injuries, the *H. longicornis* tick needs to undergo two molts in its lifetime, during which a lot of cuticle-related proteins undergo changes [25]. In the blood-sucking process of ticks, the cuticle protein begins to increase, while in the molting process, the old epidermal protein will be absorbed, and the content will decrease; at the same time, it will gradually synthesize new cuticle proteins until the end of molting. In Liu’s paper, the cuticle protein CPR1 is involved in the molting process of *H. longicornis* and is regulated by miRNA [13].

**Figure 7:**
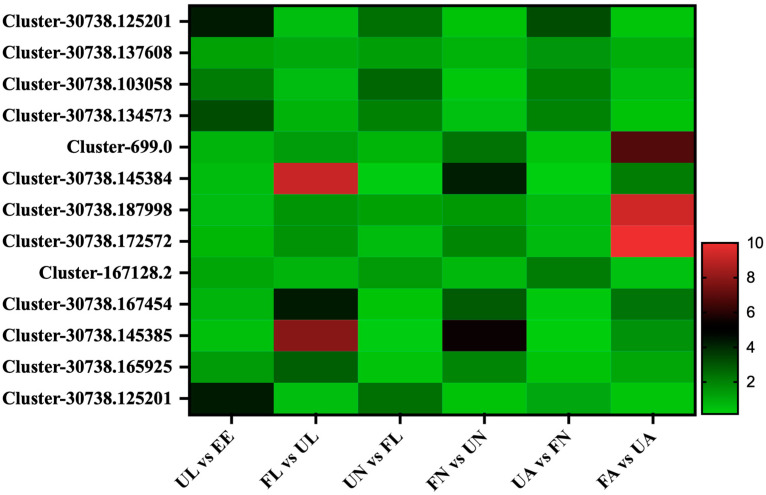
Cuticle-related proteins. EE, egg; UL, unfed larva; FL, engorged larva; UN, unfed nymph; FN, engorged nymph; UA, unfed adult; FA, engorged adult.

Thirteen cuticle proteins were found in this study. From the comparison of seven different developmental stages, the expression of these cuticle proteins showed two expression patterns: in the first pattern, Cluster-30738.125201, Cluster-30738.137608, Cluster-30738.103058, Cluster-30738.134573, Cluster-167128.2, and Cluster-30738.125201 began to increase significantly in the process of hatching from eggs to larvae. After being engorged, they showed a downward trend, and then they showed an upward trend in the process of developing into the next stage of an unfed larva. After that, this wavy mode of expression continued until the engorged adult stage. Meanwhile, the other class of cuticles had seven members, namely, Cluster-699.0, Cluster-30738.145384, Cluster-30738.187998, Cluster-30738.172572, Cluster-30738.167454, Cluster-30738.145385, and Cluster-30738.165925, which showed a downward trend in the process of egg development into the unfed larva stage. Then, in the subsequent development process, it showed a significant upward trend from the unfed stage to the corresponding engorged stage and a significant downward trend in the process from the engorged stage to the next unfed stage (Figure 7).

In this study, cuticle-associated proteins showed different expression patterns—one part showed an upward trend in the engorged stage, and the other showed a downward trend. These opposite expression trends imply that there may be great differences in the structure and function of the cuticle proteins, which need to be further analyzed in terms of their protein structure, family classification, and related functional studies.

#### 3.4.5. Membrane Proteins

A biological process is a circular network, and a membrane protein is an important hub in the network, which plays an important physiological role in organisms, such as cell proliferation and differentiation, energy conversion, signal transduction, and material transport. In addition, most drugs also achieve a therapeutic effect by interacting with membrane proteins [26].

**Figure 8:**
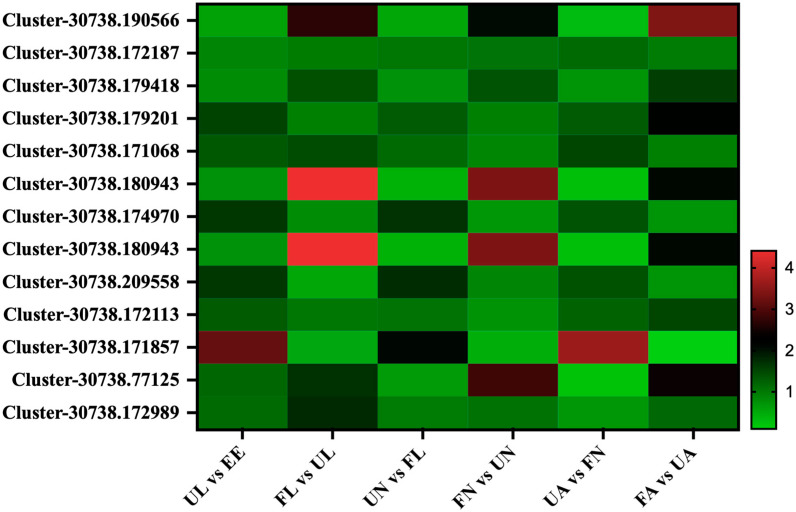
Membrane proteins. EE, egg; UL, unfed larva; FL, engorged larva; UN, unfed nymph; FN, engorged nymph; UA, unfed adult; FA, engorged adult.

A total of 12 membrane proteins were found in this study, which were divided into three patterns by the expression patterns in different developmental stages in the *H. longicornis*: the expression of the first class, Cluster-30738.172187, was relatively steady at different developmental stages, and there was no obvious stage specificity. The second class, Cluster-30738.179201, Cluster-30738.171068, Cluster-30738.174970, Cluster-30738.209558, Cluster-30738.172113, and Cluster-30738.171857, showed a significant upward trend from egg development into the unfed larva stage and then decreased significantly from the unfed stage to the corresponding engorged stage, while its expression abundance increased significantly from the engorged stage to the next unfed stage. The third class, Cluster-30738.179418, Cluster-30738.180943, Cluster-30738.180943, Cluster-30738.77125, and Cluster-30738.172989, had a common pattern, and its expression abundance increased significantly from the unfed stage to the corresponding engorged stage and reached a peak in the engorged adult stage (Figure 8).

#### 3.4.6. Salivary Proteins

The salivary gland is an important osmoregulation organ of ticks. Whether ticks are away from the host for a long time or in the feeding period of the host, the salivary glands are essential for maintaining the growth, development, and metabolism of ticks [27]. Furthermore, salivary glands and saliva play key roles in the transmission of pathogenic microorganisms to the host [28]. By using the Psiblast tool, scientists built the TickSialoFam (TSF) database, a publishable database that can help annotate tick sialo transcriptomes [29].

**Figure 9:**
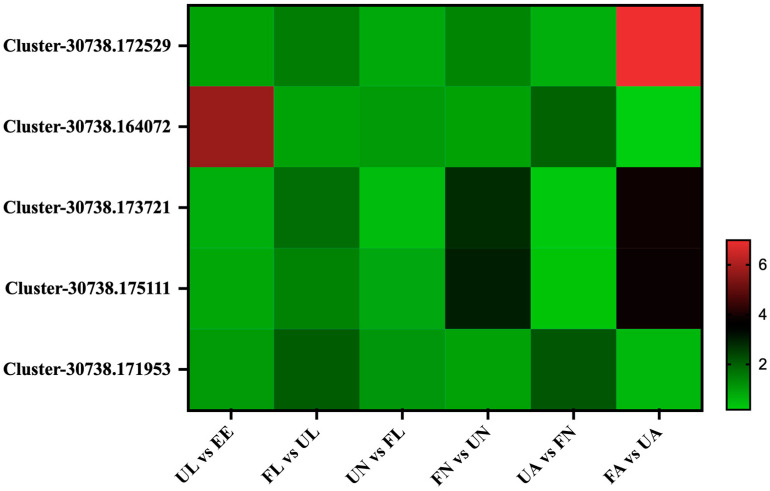
Salivary proteins. EE, egg; UL, unfed larva; FL, engorged larva; UN, unfed nymph; FN, engorged nymph; UA, unfed adult; FA, engorged adult.

Under the stimulation of blood sucking, the salivary glands will develop and enlarge rapidly, and this process will also be accompanied by changes in a large number of salivary gland-related proteins. A total of five salivary gland-associated proteins were identified in this experiment, and they were classified into two classes according to their expression patterns in seven different developmental stages. The expression abundance of the first class, Cluster-30738.172529, Cluster-30738.173721, and Cluster-30738.175111, increased rapidly in the process of blood sucking, and the expression of these proteins would continue to increase with the process of development in *H. longicornis*. The expression abundance of the second class, Cluster-30738.164072, increased rapidly in the early stage of development (eggs hatching into unfed larvae) and increased significantly during the development from unfed nymphs to engorged adults (Figure 9).

#### 3.4.7. Secreted Proteins

Secreted proteins (SP) present in parasites contribute directly or indirectly to the survival of parasites. In addition, parasites need to adapt to different hosts as well as to physiological changes during development, and SP proteins play an important role [30] in these processes.

**Figure 10:**
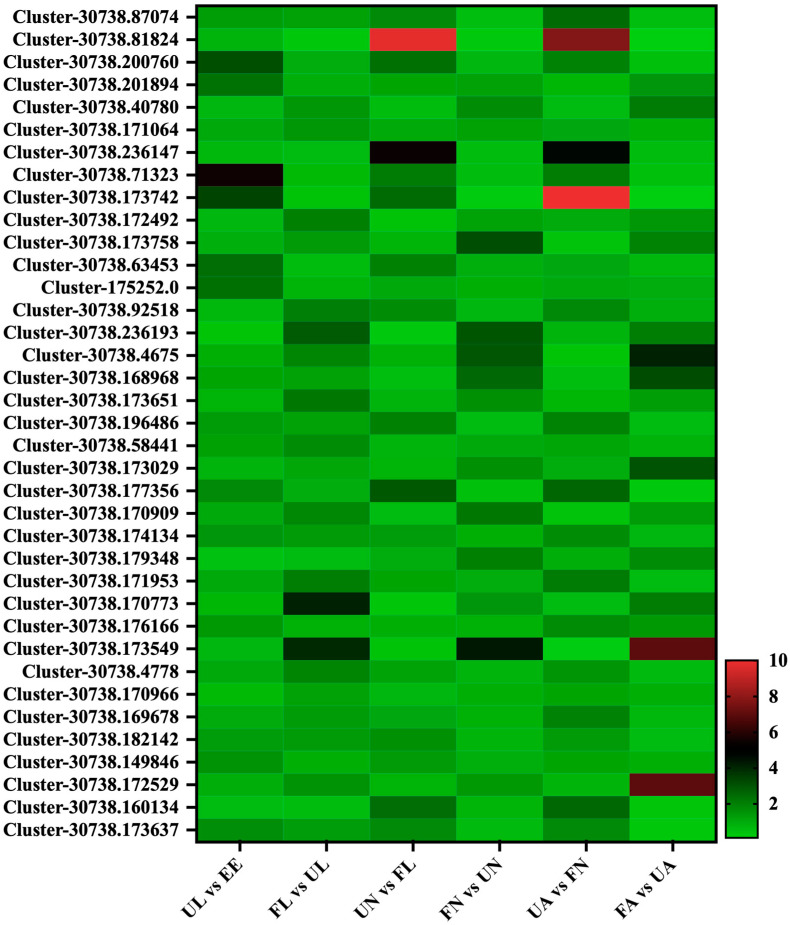
Secreted proteins. EE, egg; UL, unfed larva; FL, engorged larva; UN, unfed nymph; FN, engorged nymph; UA, unfed adult; FA, engorged adult.

In this experiment, a total of 37 secreted proteins were identified, and many proteins also showed a strong regularity and specific up-regulated expression at different developmental stages. These secreted proteins were mainly divided into three classes by collating the data. The first class, including a total of 12 proteins (Cluster-30738.87074, Cluster-30738.200760, Cluster-30738.71323, Cluster-30738.173742, Cluster-30738.63453, Cluster-175252.0, Cluster-30738.196486, Cluster-30738.177356, Cluster-30738.174134, Cluster-30738.182142, Cluster-30738.149846, and Cluster-30738.173637), showed a significant growth trend from the egg stage to the unfed larva stage. After that, its expression abundance also showed a significant growth trend in the process from the engorged stage to the next unfed stage. The second class, which consisted of 11 proteins (Cluster-30738.40780, Cluster-30738.172492, Cluster-30738.173758, Cluster-30738.236193, Cluster-30738.4675, Cluster-30738.168968, Cluster-30738.173651, Cluster-30738.173029, Cluster-30738.170909, Cluster-30738.173549, and Cluster-30738.172529), seemed to have some relationship with the process of satiety, and the expression of these proteins was significantly up-regulated in the unfed stage and the corresponding satiety stage and reached the peak at the stage of the engorged adult. In the third class, including a total of 15 proteins, the regularity of these proteins seemed to be closer to the specificity of each developmental stage of *H. longicornis*, mainly including Cluster-30738.81824, Cluster-30738.201894, Cluster-30738.171064, Cluster-30738.236147, Cluster-30738.92518, Cluster-30738.58441, Cluster-30738.179348, Cluster-30738.171953, Cluster-30738.170773, Cluster-30738.176166, Cluster-30738.4778, Cluster-30738.170966, Cluster-30738.169678, and Cluster-30738.160134 (Figure 10).

#### 3.4.8. GO Analysis of the DEPs

**Figure 11:**
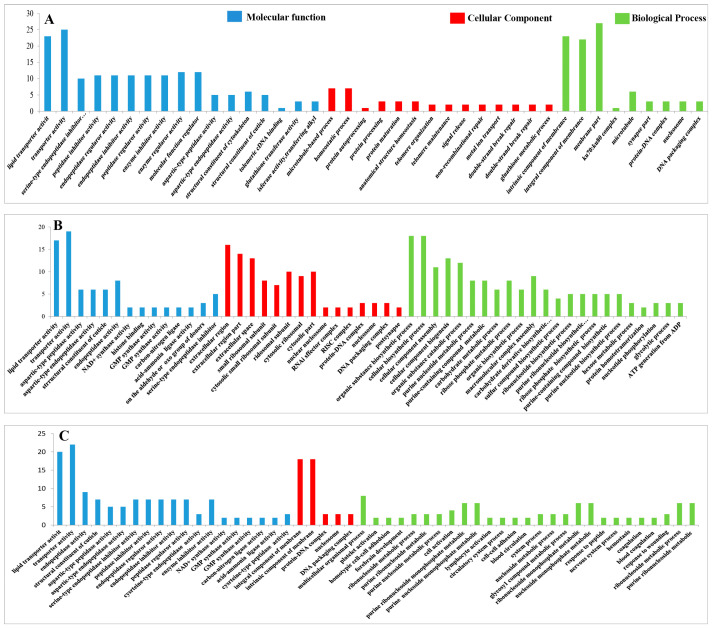
Gene Ontology (GO) enrichment for the differentially expressed proteins (DEPs) (*p* < 0.05) of the different life stages of *H. longicornis*. (**A**) Unfed larva vs. egg, (**B**) engorged larva vs. unfed larva, and (**C**) unfed nymph vs. engorged larva. GO functional annotations in the three main categories: molecular function, cellular component, and biological process.

**Figure 12:**
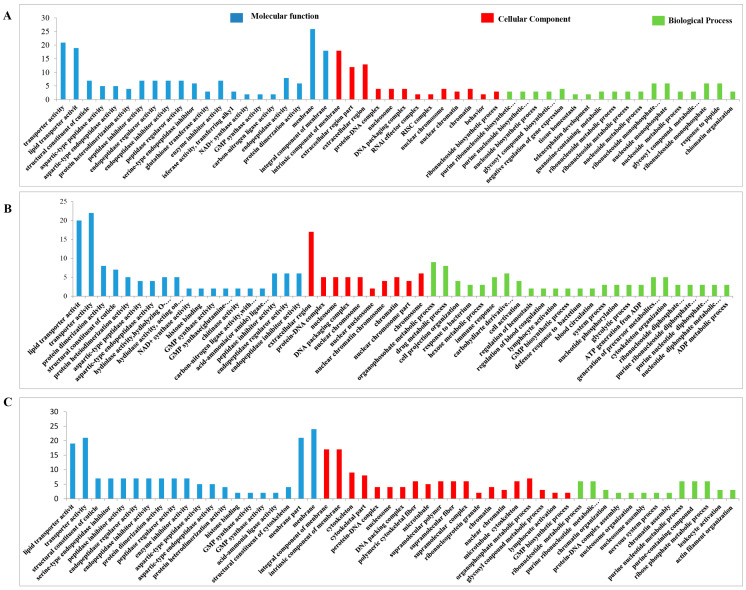
Gene Ontology (GO) enrichment for the differentially expressed proteins (DEPs) (*p* < 0.05) of the different life stages of *H. longicornis*. (**A**) Engorged nymph vs. unfed nymph, (**B**) unfed adult vs. engorged nymph, and (**C**) engorged adult vs. unfed adult. The GO functional annotations are in three main categories: molecular function, cellular component, and biological process.

The functional classification of the DEPs was carried out through GO analysis. We have identified 41, 45, 41, 44, 44, and 45 GO terms, respectively, in the UL vs. EE, FL vs. UL, UN vs. FL, FN vs. UN, UA vs. FN, and FA vs. UA groups (EE, egg; UL, unfed larva; FL, engorged larva; UN, unfed nymph; FN, engorged nymph; UA, unfed adult; FA, engorged adult). Among these GO terms, there were 19 biological process GO items, 14 cell component GO items, and 8 molecular function GO items in UL vs. EE. The GO terms in the FL vs. UL included 23 biological process terms, 14 cell component terms, and 8 molecular function terms. The GO terms in the UN vs. FL included 19 biological process terms, 14 cell component terms, and 8 molecular function terms. In FN vs. UN, there were 20 biological process terms, 14 cell component terms, and 8 molecular function terms. For the GO terms in the UA vs. FN, there were 22 biological process terms, 14 cell component terms, and 8 molecular function terms. The GO terms in FA vs. UA included 23 biological process terms, 14 cell component terms, and 8 molecular function terms. In order to further explore the functions and properties of the up-regulated and down-regulated proteins in the different developmental stages of *H. longicornis*, we performed clustering and abundance analyses of these GO terms. The figure shows up to 20 rich GO terms in each group and up to 3 main GO cluster graphs (Figure 11 and Figure 12).

Compared with eggs and unfed larva ticks, the results of the GO analysis showed the following: catalytic activity, binding, transport activity, structural molecular activity, and molecular function regulation for the molecular function; cells, cell components, organs, membrane components, and organ components for the cell composition; and the cellular process, metabolic process, regulation of biological functions, stimulus response, and recognition of cell composition/biological inheritance for the biological process.

Similarly, we also found, in the results that compared with the unfed phase, the GO analysis results in the engorged stage were as follows: the cell membrane, ribosomes, RNA-induced silencing complex (RSIC), and RNAi effector complex in the cell composition; synthesis, enzyme activity, inhibitor enzyme activity, peptidase activity, synthetase activity, and epidermal composition in the molecular function; and organic substance biosynthetic, organic substance catabolic, cofactor metabolic, cellular biosynthetic, and carbohydrate derivative metabolic in the biological processes.

In the unfed phase, compared with the previous engorged phase, the enrichment results of the GO entries were as follows: the DNA packaging complex, protein-DNA complex, nucleosome, and chromatin in the cell composition; lipid transporter activity, transporter activity, protein heterodimerization activity, a structural constituent of the cuticle, and protein kinase activity in the molecular function; and a microtubule-based process, homeostatic process, protein-DNA complex assembly, and cellular component organization in the biological process.

#### 3.4.9. KEGG Analysis of the DEPs

**Figure 13:**
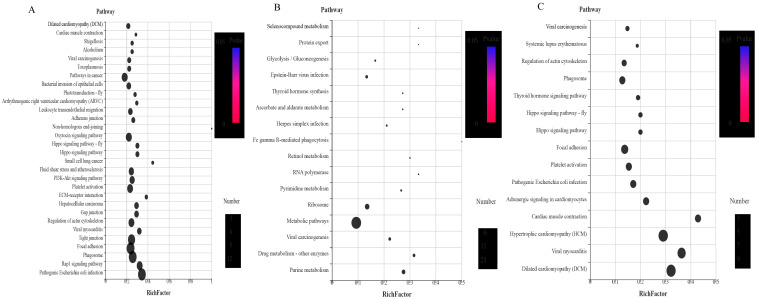
Kyoto Encyclopedia of Genes and Genomes (KEGG) enrichment analysis of the differentially expressed proteins (DEPs) (*p* < 0.05) of the different life stages of *H. longicornis*. *p* < 0.05 indicates significant enrichment in the development-related pathways. The top 20 pathways are shown. (**A**) Unfed larva vs. egg, (**B**) engorged larva vs. unfed larva, and (**C**) unfed nymph vs. engorged larva. The KEGG enrichment was measured by the Rich factor, *q*-value, and the number of genes enriched in this pathway. The colors and sizes of the spots represent the *q*-values and the number of target genes, respectively. EE, egg; UL, unfed larva; FL, engorged larva; UN, unfed nymph.

**Figure 14:**
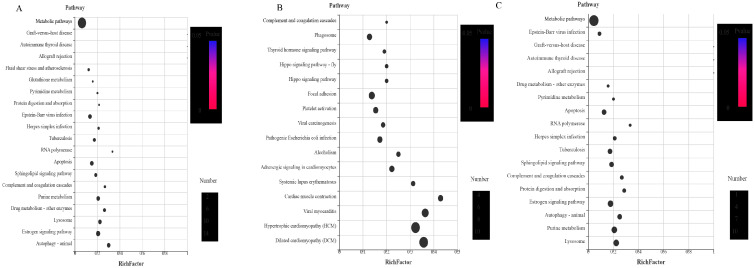
Kyoto Encyclopedia of Genes and Genomes (KEGG) enrichment analysis of the differentially expressed proteins (DEPs) (*p* < 0.05) of the different life stages of *H. longicornis*. *p* < 0.05 indicates significant enrichment in the development-related pathways. The top 20 pathways are shown. (**A**) engorged nymph vs. unfed nymph, (**B**) unfed adult vs. engorged nymph, and (**C**) engorged adult vs. unfed adult. The KEGG enrichment was measured by the Rich factor, *q*-value, and number of genes enriched in this pathway. The colors and sizes of the spots represent the *q*-values and the number of target genes, respectively. UN, unfed nymph; FN, engorged nymph; UA, unfed adult; FA, engorged adult.

In order to further determine the biological pathways in which these differential proteins are involved in the development of *H. longicornis*, the UL vs. EE, FL vs. UL, UN vs. FL, FN vs. UN, UA vs. FN, and FA vs. UA groups (EE, egg; UL, unfed larva; FL, engorged larva; UN, unfed nymph; FN, engorged nymph; UA, unfed adult; FA, engorged adult) were analyzed and identified 2112, 2124, 4323, 3371, 2846, and 1998 channels, respectively (Figure 13 and Figure 14).

Among these pathways, it was found that, compared with the unfed stage, the signal pathways enriched by the up-regulated proteins in the engorged stage mainly included the digestive system, immune system, endocrine system, environmental adaptation, and infectious diseases (viral, and bacterial), signal transduction, cellular community eukaryotes, cell growth and death, and transport and catabolism.

In the unfed stage, compared with the previous engorged stage, the main enriched signal pathways included the excretory system, nervous system, aging, development, cardiovascular diseases, folding, sorting and degradation, replication and repair, and cell motility.

## 4. Conclusions

This is the first in-depth overview of the protein spectrum of *H. longicornis* (parthenogenesis), which could be of great significance for revealing the molecular architecture of ticks with a complex life cycle. Our data provide strong molecular support for the use of *H. longicornis* as a powerful model for studying tick development and reveal a group of proteins. These proteomes have expanded to play a key role in biological regulatory processes such as digestion, molting, ovarian development, and immunomodulation. Overall, this is a report on the overall proteomics of *H. longicornis*, which will help us to understand the complex process of tick development, and the membrane-associated proteins and secretory proteins described in this paper will also help to find new target proteins and provide a theoretical basis and candidates for improving tick control strategies.

## Figures and Tables

**Table 1:** Primers of the six genes for quantitative reverse transcription polymerase chain reaction.

Gene	Primer Name	Primer Sequence (5′–3′)
Flotillin	Flotillin F	CCATCAAGGACATCAGCGAT
	Flotillin R	TATGCAGCCTTCTTGAGCTC
Mo-25	Mo-25 F	GCACACGTTCACCAACAGTA
	Mo-25 R	ACAAGATCGCAATCCTCCTG
Dystrophin	Dystrophin F	AACGTGCACGTGTCCATGC
	Dystrophin R	GAGGTTCTGCAGGAGGTTGA
CRK	CRK F	AGAACGAAGTCGGCGTCTTT
	CRK R	ATGTCGGGGAACATCTGGT
Septin-1	Septin-1 F	TGTTATCAGCATGGTCCTGA
	Septin-1 R	AAGCAGCTCAAGGAGTCCGT
Septin-2	Septin-2 F	AACAGCTTGTCAGCAAATC
	Septin-2 R	GACGTTGCCCTCTTGCAATT
β-actin	β-actin F	CGTTCCTGGGTATGGAATCG
	β-actin R	TCCACGTCGCACTTCATGAT

**Table 2:** Spectra, peptides, and proteins identified by the isobaric tags for relative and absolute quantification (iTRAQ).

	Total-Spectra	Spectra	Peptide	Unique-Peptide	Protein-Identified
Repeat 1	839,950	7932	2769	2663	1353
Repeat 2	829,948	8019	2781	2673	1323
Repeat 3	938,964	8951	2939	2844	1437
Total	2,608,862	24,902	4405	4295	2044

## Data Availability

The data reported in this paper have been deposited in the OMIX, China National Center for Bioinformation/Beijing Institute of Genomics, Chinese Academy of Sciences “https://ngdc.cncb.ac.cn/omix/release/OMIX707 (accessed on 25 October 2021)”.

## Supplementary Materials

The following supporting information can be downloaded at: https://www.mdpi.com/article/10.3390/life15010059/s1, Table S1: The details of 2044 proteins that were identified in this study.
